# Identification of new members of the *Escherichia coli* K-12 MG1655 SlyA regulon

**DOI:** 10.1099/mic.0.000423

**Published:** 2017-03-29

**Authors:** Thomas D Curran, Fatima Abacha, Stephen P Hibberd, Matthew D Rolfe, Melissa M Lacey, Jeffrey Green

**Affiliations:** ^1^​ Department of Molecular Biology and Biotechnology, University of Sheffield, Sheffield, S10 2TN, UK; ^2^​ Biomolecular Research Centre, Sheffield Hallam University, Sheffield, S1 1WB, UK

**Keywords:** biofilm, gene expression, MarR family, transcription regulation

## Abstract

SlyA is a member of the MarR family of bacterial transcriptional regulators. Previously, SlyA has been shown to directly regulate only two operons in *Escherichia coli* K-12 MG1655, *fimB* and *hlyE* (*clyA*). In both cases, SlyA activates gene expression by antagonizing repression by the nucleoid-associated protein H-NS. Here, the transcript profiles of aerobic glucose-limited steady-state chemostat cultures of *E. coli* K-12 MG1655, *slyA* mutant and *slyA* over-expression strains are reported. The transcript profile of the *slyA* mutant was not significantly different from that of the parent; however, that of the *slyA* expression strain was significantly different from that of the vector control. Transcripts representing 27 operons were increased in abundance, whereas 3 were decreased. Of the 30 differentially regulated operons, 24 have previously been associated with sites of H-NS binding, suggesting that antagonism of H-NS repression is a common feature of SlyA-mediated transcription regulation. Direct binding of SlyA to DNA located upstream of a selection of these targets permitted the identification of new operons likely to be directly regulated by SlyA. Transcripts of four operons coding for cryptic adhesins exhibited enhanced expression, and this was consistent with enhanced biofilm formation associated with the SlyA over-producing strain.

## Introduction

The MarR family of transcription regulators are widespread throughout the Bacterial and Archeal kingdoms [1]. MarR family members are homodimeric and bind to palindromic DNA sequences within regulated promoters using a characteristic winged-helix-turn-helix DNA binding domain [2]. These regulators repress gene expression by promoter occlusion (e.g. MarR [3]) or activate gene expression by either stabilizing RNA polymerase/promoter DNA interactions (e.g. OhrR [4]) or antagonizing the action of repressors (e.g. RovA [5]). These activities of MarR proteins are inhibited upon interaction with cognate signalling molecules, although for many members the natural ligand is unknown [6].

The *Salmonella enterica* serovar Typhimurium LT2 SlyA protein is one of the best characterized members of the MarR family. The *S. enterica* serovar Typhimurium *slyA* mutant is attenuated for virulence, is hypersensitive to oxidative stress and is impaired for survival in macrophages [7, 8]. A consensus DNA binding site has been proposed, TTAGCAAGCTAA [9, 10], and proteomic and transcriptomic comparisons of parent and *slyA* mutant strains suggest that SlyA can act as both a negative and a positive regulator of gene expression, with significant overlap with genes of the PhoPQ regulon involved in cell envelope function, virulence, resistance to anti-microbial peptides and regulation of small RNAs [11–15]. *S. enterica* serovar Typhimurium 14028s SlyA has also been linked to the stringent response by binding ppGpp resulting in enhanced DNA binding [16, 17]. The expression of many SlyA-regulated genes is subject to H-NS-mediated silencing, and activation of these genes generally involves an element of antagonism of H-NS repression by SlyA [11, 18–22].

The SlyA protein of *Escherichia coli* MG1655 is 91 % identical and 95 % similar (over 142 amino acids) to the *S. enterica* serovar Typhimurium LT2 protein, but it is much more poorly characterized. Only two genes, *hlyE* and *fimB* (as well as auto-regulation of *slyA*), have been shown to be directly regulated by SlyA [19, 21, 23]. In some other *E. coli* strains, SlyA regulates capsule synthesis and lipid A palmitoylation in biofilms [18, 19, 22]. Here, transcriptional profiling of parent, *slyA* mutant and *slyA* over-expression strains reveals the breadth of the *E. coli* MG1655 SlyA regulon, indicating roles in activating expression of cryptic fimbrial-like adhesins that contribute to enhanced biofilm formation.

## Methods

### Bacterial strains, plasmids, oligonucleotides and growth conditions

The bacterial strains, plasmids and oligonucleotides that were used are listed in Table 1. Bacterial strains were routinely cultured in LB broth or on LB agar plates [24]. Aerobic glucose-limited steady-state chemostat cultures of *E. coli* were established in Evans minimal medium [25] in Labfors 3 fermentation vessels (Infors HT) with a 1 l working volume, 0.2 h^−1^ dilution rate, 37 °C, pH 6.9, 400 r.p.m. stirring rate and sparging with 1 l min^−1^ air. Evans minimal medium consists of the following: 10 mM NaH_2_PO_4_, 10 mM KCl, 1.25 mM MgCl_2_, 20 mM NH_4_Cl, 0.02 mM CaCl_2_, 0.1 mM Na_2_SeO_3_, 1.5 mM monosodium nitrilotriacetate, 20 mM glucose and 100 ml trace element solution. The trace element solution consisted of the following (g l^−1^): ZnO (0.412), FeCl_3_·6H_2_O (5.4), MnCl_2_·4H_2_O (2.0), CuCl_2_·2H_2_O (0.172), CoCl_2_·6H_2_O (0.476), H_3_BO_3_ (0.064) and Na_2_MoO_4_·H_2_O (0.004) in 0.3 % (v/v) HCl. For generation of cell paste for purification of His-tagged SlyA, *E. coli* BL21 (λDE3) transformed with pGS2469 was grown in auto-induction medium supplemented with ampicillin (100 mg l^−1^) [26]. Resistance to chloramphenicol was tested by inoculating LB broth (2 ml) containing kanamycin (30 µg ml^−1^) and 0, 1, 2, 3 or 4 µg ml^−1^ chloramphenicol with 10 µl of overnight starter cultures (*E. coli* K-12 MG1655 pET28a or *E. coli* K-12 MG1655 pGS2468). Triplicate cultures were grown under aerobic conditions for 6 h at 37 °C before measuring OD_600_ as an indicator of growth. The experiment was carried out twice.

**Table 1.** Bacterial strains, plasmids and oligonucleotides

Strain or plasmid	Relevant characteristics*	Reference or source
Bacterial strain		
*E. coli* BL21 (λDE3)	*E. coli* BL21 lysogen for inducible (IPTG) expression of the T7 RNA polymerase	Novagen
*E. coli* JRG6457	*E. coli* MG1655 *slyA*	This work
*E. coli* JRG6636	*E. coli* MG1655 pGS2468	This work
*E. coli* JRG6072	*E. coli* MG1655 pKD46	This work
*E. coli*	Genome-sequenced parental strain MG1655	[47]
Plasmid		
pET28a	Multi-copy plasmid; Kan^R^	Novagen
pGS2468	pET28a derivative for expression of *slyA* from the *slyA* promoter; Kan^R^	This work
pGS2469	pLATE-51 derivative for overproduction of SlyA; Amp^R^	This work
pKD4	Source of kanamycin resistance cassette; Amp^R^, Kan^R^	[48]
pKD46	Plasmid for inducible (l-arabinose) expression of the λred recombinase; Amp^R^, T^s^	[48]
pLATE-51	Expression vector for production of His-tagged proteins; Amp^R^	Thermo Scientific
Oligonucleotide		
TC7	TAAAGCCGCATAATATCTTAGCAAGCTAATTATAAGGAGATTACACGTCTTGAGCGATT; creation of *slyA* mutant	This work
TC8	TTGCGTGTGGTCAGGTTACTGACCACACGCCCCCTTCATTCATATGAATATCCTCCTTAG; creation of *slyA* mutant	This work
TC9	CTGACGGTAACCAAATGCAG; PCR of *slyA* locus	This work
TC10	TTTGCGTGTGGTCAGGTTAC; PCR of *slyA* locus	This work
TC49	[Btn]ACTCTCTCCTTATAACCAATTG; forward primer for PCR of biotin (Btn)-labelled 355 bp intergenic region between *ssuE* and *elfA*	This work
TC50	CGTTATCATCCTGATCTCTT; reverse primer for use with TC49	This work
TC51	[Btn]TGGTGAATATTATTGATCAATTAAT; forward primer for PCR of biotin (Btn)-labelled 344 bp intergenic region between *leuO* and *leuL*	This work
TC52	ACTTAACTCCACTGTCACACTTAA; reverse primer for use with TC51	This work
TC53	[Btn]TTGTTCTCCTTCATATGCTC; forward primer for PCR of biotin (Btn)-labelled 414 bp intergenic region between *casA* and *cas3*	This work
TC54	CTTCGGGAATGATTGTTATC; reverse primer for use with TC53	This work
TC55	[Btn]TGTTGCTAATAGTTAAATCGC; forward primer for PCR of biotin (Btn)-labelled 257 bp intergenic region between *paaA* and *paaZ*	This work
TC56	GTCATCACCTTTACGATTCC; reverse primer for use with TC55	This work
TC57	[Btn]AACAAACAACTCCTTGTCCG; forward primer for PCR of biotin (Btn)-labelled 400 bp region upstream of *mdtM*	This work
TC58	CCCCGAGGCGCTTTCCAGGC; reverse primer for use with TC57	This work
TC59	[Btn]AGAACTTCCTGTTTTAATTATTG; forward primer for PCR of biotin (Btn)-labelled 179 bp intergenic region between *gspA* and *gspC*	This work
TC60	GATGTATGTTCTAATAAAATAGATTG; reverse primer for use with TC59	This work
TC61	[Btn]CCGTCGTTGACTCCATGC; forward primer for PCR of biotin (Btn)-labelled 130 bp intergenic region between *sgcA* and *sgcQ*	This work
TC62	GATGGGGATAAGCAGAGC; reverse primer for use with TC61	This work
TC63	[Btn]GCGGAGTGCATCAAAAGT; forward primer for PCR of biotin (Btn)-labelled 291 bp intergenic region between *fecI* and *insA-7*	This work
TC64	GCAAGCACCTTAAAATCAC; reverse primer for use with TC63	This work
TC65	[Btn]TTTCATCTCCTTATAATTAGCTT; forward primer for PCR of biotin (Btn)-labelled 200 bp intergenic region between *slyA* and *ydhI*	This work
TC66	AAAGTAGATTCCTTTACGACC; reverse primer for use with TC65	This work
TC70	[Btn]AGCTATCTCCGTAGACCGT; forward primer for PCR of biotin (Btn)-labelled 400 bp region upstream of *sgcX*	This work
TC71	GATTATCTATACTCCCTCTGAATC; reverse primer for use with TC70	This work

*Amp^R^, ampicillin resistant; Kan^R^, kanamycin resistant; T^s^, temperature-sensitive replication.

### Biofilm assay

Biofilm assays were performed using 96-well plates essentially as described by Tagliabue *et al.* [27] using M9 minimal medium with 20 % (w/v) glucose and 50 µg ml^−1^ kanamycin. Wells containing 200 µl of medium were inoculated (1 : 10) from an overnight culture of *E. coli* K-12 MG1655 pET28a or *E. coli* K-12 MG1655 pGS2468 and then incubated for 16 h under aerobic conditions at 37 °C. Growth of cultures was monitored by measuring OD_600_. The planktonic cells were removed and the remaining biofilm was stained for 5 min with 200 µl of 1 % (w/v) crystal violet solution. Excess stain was removed by three washes with deionized water before the plate was air-dried. To quantify the extent of staining, 200 µl ethanol : acetone (4 : 1) was added to each well, and after incubating for 20 min, the amount of biofilm was estimated by measuring *A*_600_. Adhesion units were calculated by dividing the *A*_600_ values for crystal violet-stained adhered cells by the OD_600_ values for the corresponding planktonic cells.

### Creation of *E. coli* K-12 MG1655 *slyA* mutant

A PCR-amplified DNA fragment containing the kanamycin cassette from pKD4 flanked by 40 bp DNA homologous to regions surrounding the *slyA* gene was synthesized using oligonucleotide primers TC7 and TC8 (Table 1). The purified (QIAquick PCR cleanup, Qiagen) PCR product (5 µg) was used to transform *E. coli* JRG6072 by electroporation (Hybaid Cell Shock unit; 1800 V, 1 mm path length). The *E. coli* JRG6072 competent cells were prepared from aerobic LB broth batch cultures supplemented with ampicillin (100 mg l^−1^) at 30 °C that had been induced to express the λ red recombinase by addition of l-arabinose (1 mM). Kanamycin-resistant mutants were selected on LB agar plates containing kanamycin (30 mg l^−1^) at 37 °C. Mutation of the *slyA* gene by insertion of the kanamycin resistance cassette was confirmed by colony PCR using oligonucleotides TC9 and TC10. The *slyA* mutation was then transduced using bacteriophage P1 to *E. coli* MG1655 [24].

### Transcriptional profiling

Transcriptomic analyses were carried out as described by Rolfe *et al.* [28] using directly quenched samples from glucose-limited steady-state chemostat cultures (dilution rate=0.2 h^−1^) for the three *E. coli* K-12 MG1655 strains: parent, *slyA* mutant (JRG6457) and *slyA* over-producer (JRG6636). RNA samples were labelled with Cy5 and the reference *E. coli* K-12 MG1655 genomic DNA was labelled with Cy3. In total, two independent biological replicates were performed that were hybridized in duplicate (technical replicates), giving four replicates. After hybridization and image capture, data were extracted from the raw image files using Agilent Feature Extraction v11.5 software and analysed using GeneSpring v7.3.1. Transcriptomic data have been deposited with ArrayExpress (accession E-MTAB-5220).

### Purification of SlyA and Western blotting

Cultures (500 ml auto-induction medium supplemented with ampicillin in 2 l conical flasks) of *E. coli* BL21 (λDE3) pGS2469 were grown at 37 °C for 24 h with shaking (250 r.p.m.). Bacteria were collected by centrifugation, the pellet was re-suspended in 15 ml of breakage buffer [20 mM Tris/HCl, 500 mM NaCl and 5 % (v/v) glycerol, pH 7.5], the bacteria were lysed by two passages through a French pressure cell (16 000 psi) and the extract was clarified by centrifugation (27 000***g***, 15 min, 4 °C). The His-tagged SlyA protein was isolated from the cell-free extract by affinity chromatography on a HiTrap chelating column (1 ml) attached to an AKTA prime according to the standard manufacturer’s protocol (GE Healthcare). The eluted SlyA was buffer exchanged into 20 mM Tris/HCl, pH 7.4, containing 200 mM NaCl by repeated dilution and Vivaspin 6 concentration (Sartorius Stedim Biotech). The protein was judged to be >90 % pure by Coomassie blue-stained SDS-PAGE, and protein concentration was estimated by the BioRad protein reagent protocol [29]. SlyA protein was detected by Western blotting after separation of polypeptides by SDS-PAGE and electrophoretic transfer [100 V for 1 h; transfer buffer: 5.8 g l^−1^ Tris, 2.9 g l^−1^ glycine, 20 % (v/v) methanol and 0.037 % (w/v) SDS] to Hybond-C extra nitrocellulose membranes (GE Healthcare). The membranes were soaked in a blocking solution, which contained 5 % (w/v) dried skimmed milk in PBS (10 mM phosphate buffer, 137 mM NaCl and 2.7 mM KCl, pH 7.4) and 0.05 % (v/v) Tween 20, for 16 h at 4 °C. The blocking solution was then removed and the membranes were washed in PBS containing 0.05 % (v/v) Tween 20 before exposure to a 1 : 1000 dilution of the SlyA antibody (raised in rabbit and provided by Prof. Ian Blomfield, University of Kent) in blocking solution for 1 h at room temperature. After four washes with PBS containing 0.05 % (v/v) Tween 20, the membranes were soaked in blocking solution containing anti-rabbit secondary antibody provided in the Pierce ECL Western Blotting kit, and the presence of SlyA was visualized according to the manfacturer’s standard protocol (Thermo Scientific).

### Electrophoretic mobility shift assays (EMSA)

The LightShift Chemiluminescent EMSA kit (Thermo Scientific) was used according to the manufacturer’s instructions. Biotin-labelled DNA of target promoter regions was amplified from genomic DNA using the appropriate oligonucleotide primer pairs (Table 1). The core binding assays (20 µl) contained 2 µl of 10× binding buffer [100 mM Tris/HCl, pH 7.5, containing 500 mM KCl and 10 mM dithiothreitol and 1 µg poly (dI•dC)]. The DNA concentration was ~1 nM and the concentration of SlyA ranged from 0 to 500 nM as indicated. Mixtures were incubated at 25 °C for 30 min before separation of SlyA/DNA complexes by native gel electrophoresis, followed by transfer to Hybond-N+ nylon membranes, UV crosslinking for 60 s at 120 mJ cm^−2^ and detection of labelled DNA using the Nucleic Acid Detection Module (Thermo Scientific).

## Results and discussion

### Enhanced expression of *slyA* in *E. coli* K-12 MG1655 results in altered abundance of transcripts from 30 operons

Previous work has shown that SlyA directly activates the expression of two genes in *E. coli* K-12 (*hlyE* and *fimB*) by antagonizing H-NS repression [20, 21, 23]. However, in *S. enterica* serovar Typhimurium, the influence of SlyA is much more extensive, with at least 31 regulated genes resulting in hypersensitivity to reactive oxygen species and attenuation in infection models [7, 8, 12]. The initial aim of this work was to apply transcript profiling to determine the extent of the *E. coli* K-12 MG1655 SlyA regulon by comparison of steady-state glucose-limited aerobic chemostat cultures of wild-type and *slyA* mutant strains. Comparison of transcript profiles of wild-type and *slyA* mutant cultures grown at a dilution rate of 0.5 h^−1^ (equivalent to a doubling time of 1.4 h) revealed no significant (≥2-fold; *P*≤0.05) changes in transcript abundance. Because SlyA translation may be enhanced at low growth rates, due to its unusual UUG start codon [21], steady-state cultures at dilution rates 0.2, 0.1 and 0.05 h^−1^ were established (equivalent to doubling times of 3.5, 6.9 and 13.8 h, respectively). However, once again, when the transcript profiles and growth characteristics of the wild-type and *slyA* mutant cultures were compared, no significant differences were detected. These observations indicated that, under the conditions tested, deletion of the *slyA* gene had no significant effect on gene expression in *E. coli* K-12 MG1655, even at low growth rates.

**Fig. 1.**
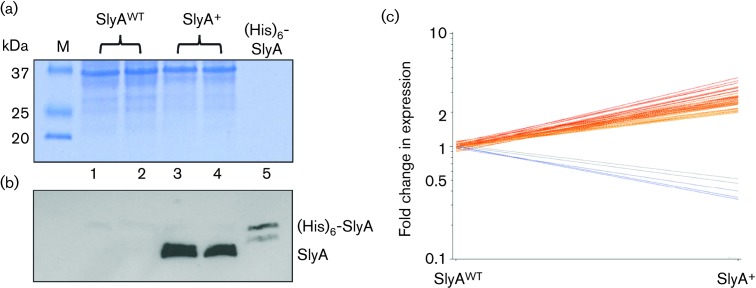
Changes in the transcript profile of *E. coli* K-12 MG1655 over-producing SlyA. Transformation of *E. coli* K-12 MG1655 with a multi-copy plasmid expressing *slyA* under the control of its own promoter results in detectable SlyA protein in lysed cell suspensions from aerobic steady-state glucose-limited chemostat cultures. The upper panel (a) shows the Coomassie blue-stained SDS-polyacrylamide gel and the lower panel (b) shows the relevant region of a Western blot prepared with the same samples and loadings developed with SlyA antiserum. The gels were loaded as follows: lane M, SDS-PAGE markers (sizes, kDa, are indicated); lanes 1 and 2, extracts from independent cultures of *E. coli* K-12 MG1655 transformed with the vector pET28a (SlyA^WT^); lanes 3 and 4, extracts from independent cultures of *E. coli* K-12 MG1655 transformed with the expression plasmid pGS2468 (SlyA^+^); lane 5, purified (His)_6_-SlyA (~10 ng protein loaded). (b) Western blot corresponding to the gel shown in (a). The locations of SlyA and purified (His)_6_-SlyA are indicated. (c) Graphical representation of the changes in transcript abundance occurring upon over-production of SlyA in *E. coli* K-12 MG1655. Comparison of the fold changes in transcript abundance of aerobic steady-state glucose-limited chemostat cultures of *E. coli* K-12 MG1655 transformed with either pET28a (SlyA^WT^) or pGS2468 (SlyA^+^). Each line represents a gene that exhibits a ≥2-fold change in transcript abundance (*P*≤0.05) from two biological and two technical replicates, i.e. four measurements.

Anti-SlyA serum was used to determine whether SlyA was detectable in *E. coli* K-12 MG1655 cells grown in glucose-limited chemostats at a dilution rate of 0.2 h^−1^. In accordance with the transcript profiling, SlyA was not detected (Fig. 1). This suggests that the expression of SlyA is regulated and switched on under conditions other than those imposed here; for example, SlyA protein has been detected by Western blotting extracts from *E. coli* batch cultures grown in minimal medium with glycerol as the carbon and energy source [21]. To overcome any regulatory barrier to identifying genes potentially controlled by SlyA, a plasmid (pGS2468) to express *slyA* under the control of its own promoter was constructed. Western blotting showed that SlyA protein was now readily detectable in the transformed *E. coli* K-12 MG1655 cells grown in glucose-limited chemostats at a dilution rate of 0.2 h^−1^ (Fig. 1). The growth characteristics of the vector control and the *slyA* expression strains were essentially the same, with similar yields (1.4±0.2 g cell dry weight per litre) and no detectable glucose or over-metabolites in the culture supernatants. Therefore, the transcript profiling experiments were carried out with these strains grown in aerobic glucose-limited chemostats at a dilution rate of 0.2 h^−1^. The transcript profile of the SlyA over-production strain was significantly different from that of the vector control. Transcripts representing 27 operons were increased in abundance and 3 were decreased (Table 2; Fig. 1c). The transcripts exhibiting decreased abundance were the *sgc* operon (*sgcXBCQAER*), which encodes a phosphotransferase system for the uptake of an unknown sugar; *fecIR*, the membrane-bound sensor (FecR) that receives signals from the outer membrane ferric citrate uptake receptor (FecA) for transmission to FecI (σ^19^), which activates transcription of the *fecABCDE* operon encoding components of a cytoplasmic membrane-bound ferric citrate uptake system; and *yecH*, which encodes a predicted protein of unknown function (Table 2) [30, 31].

**Table 2.** Transcripts exhibiting altered abundance upon over-expression of *slyA* in *E. coli* MG1655

Operon*	Fold change†	Gene function‡	H-NS regulon§	LeuO regulon||	Overlap with genomic island¶
*ybeT*	4.1	Conserved outer membrane protein	K	✓	
*trkG*	3.8	Rac prophage potassium transporter subunit	K, O		IV
*ssuE**A**DCB*	3.6	Aliphatic sulfonate transport and metabolism	G, K, O	✓	
*yehDCBA*	3.6	Chaperone-usher fimbrial operon (cryptic)	K, O	✓	GIST
*mngAB*	3.4	2-*O*-α-Mannosyl-d-glycerate PTS and α-mannosidase		✓	
*casABC*	3.3	CRISPR-associated genes	K	✓	GIST, IV
*yghS*	3.1	Predicted protein with nucleoside triphosphate hydrolase domain	K, O	✓	
*slyA*	3.0	DNA-binding transcriptional activator	O		
*yfbN*	2.8	Predicted protein	K, O	✓	IV
*paaA-K*	2.8	Phenylacetic acid degradation			
*ybeU-hscD*	2.8	Predicted tRNA ligase and chaperone	K, O	✓	
*elfADCG-ycbUVF*	2.7	Predicted fimbrial-like adhesin protein (cryptic)	G, K, O	✓	
*ygeG*	2.7	Predicted chaperone	G, K, O		GIST
*crfC-yjcZ*	2.6	Clamp-binding sister replication fork co-localization protein and predicted protein	K, O		
*sfmHF*	2.6	Predicted fimbrial-like adhesin protein (cryptic)	O		IV
*agaS-kbaY-aga**B**CDI*	2.5	Predicted galactosamine transport and metabolism (cryptic)		✓	
*ydhY**V**-T*	2.5	Predicted oxidoreductase	G, K, O		GIST
*yiiE*	2.5	Predicted transcriptional regulator	K, O		
*mdtM*	2.5	Multi-drug efflux system protein			
*leuO*	2.5	DNA-binding transcriptional activator	G, K, O	✓	GIST
*C0299*	2.4	sRNA C0299	O		
*ycjM**N**-V*	2.4	Predicted sugar transporter and metabolism	K, O	✓	
*yadN*	2.4	Predicted fimbrial-like adhesin protein (cryptic)	G, K, O	✓	GIST
*gspCDEF*	2.4	Type II secretion system (cryptic)	K, O	✓	
*ydhIJK*	2.2	Predicted proteins	O		
*yfdM*	2.1	CPS-53 (KpLE1) prophage predicted methyltransferase	O		GIST, IV
*hlyE*	2.0	Hemolysin E (cryptic)	K, O		
*yecH*	0.5	Predicted protein	O		GIST
*sgcX**B**CQAER*	0.5	Predicted sugar transport and metabolism			IV
*fecI**R***	0.4	Transcription regulation of ferric citrate transport			IV

*The fold change data shown are for the first gene in the operon except where indicated by bold typeface; note that all genes in the operons followed the same pattern of regulation.

†Fold change (≥2-fold, *P*≤0.05) is the product of dividing the transcript abundance for the *slyA* over-expression cultures by that for the control cultures.

‡Gene functions as assigned in Ecocyc.org [46].

§Genes associated with H-NS binding were identified from Grainger *et al*. [49] (G), Kahramanoglou *et al*. [50] (K) and Oshima *et al*. [51] (O).

||Genes located upstream or downstream of a LeuO binding site identified by Shimada *et al*. [44].

¶Genes the overlap with genomic islands in *E. coli* K-12 MG1655 identified by GIST and/or IslandViewer (IV) [34].

Among the up-regulated transcripts were the previously identified SlyA-regulated gene *hlyE* and *slyA* itself (Table 2). The latter finding was not surprising as the *slyA* gene was present in multi-copy, but despite this, the *slyA* transcript only increased ~3-fold in abundance, yet the SlyA protein level increased from being undetectable in the control to a level equivalent to ~1.5 µM in the cytoplasm (based on the dry weight of *E. coli* being 3×10^−13^ g with an aqueous volume of 7×10^−13^ ml per cell [32]). The relatively low level of induction of the *slyA* transcript when present in multi-copy but much greater induction of SlyA protein suggests that the *slyA* promoter is subject to auto-regulation, consistent with the reported SlyA binding at the *slyA* promoter [19]. It was also notable that the *ydhI-K* operon, which is divergently transcribed from *slyA* and not present on the *slyA* expression plasmid, also exhibited enhanced transcript abundance, suggesting that SlyA is capable of activating expression from divergent promoters, an assertion supported by the enhanced abundances of the divergently transcribed *hlyE* and *C0299* (encodes a small RNA) genes in the presence of SlyA (Table 2).

Of the 30 operons that showed altered transcript abundance upon over-production of SlyA, 24 (~80 %) have also been shown to be associated with H-NS binding sites (Table 2). Thus, it appears that H-NS-repressed genes are over-represented in the set of transcripts that increase in abundance when SlyA is expressed, suggesting that SlyA acts by antagonizing H-NS repression at the corresponding promoters, a mechanism that is established for *hlyE* [20]. H-NS binds DNA by recognizing the structure of A-T-rich minor grooves and silences the expression of horizontally acquired A-T-rich genes (reviewed by Navarre [33]). H-NS is thus considered crucial in permitting the acquisition of new genes while counteracting the potentially detrimental effects of inappropriate expression of these genes. Counter-silencing by H-NS antagonists, such as SlyA, provides a route to integrate expression of the genes into the regulatory circuits of *E. coli* under appropriate conditions. Horizontally acquired genes are located within genomic islands, which are regions of bacterial chromosomes that are often associated with drug resistance, metabolic adaptability, stress tolerance and pathogenesis. Genomic islands can be recognized by their sequence composition and increased transcript start point densities [32]. The analysis tools GIST (Genomic-island Identification by Signals of Transcription) and IslandViewer have been used to map the genomic islands of *E. coli* K-12 MG1655 [34]. Notably, 13 of the 30 differentially regulated operons overlapped predicted genomic islands, suggesting a general role for SlyA in the counter-silencing of H-NS-repressed horizontally acquired genes under conditions when *slyA* is up-regulated (Table 2).

The H-NS-repressed *casABC* operon was up-regulated by SlyA (Table 2). This operon encodes proteins involved in maintaining and utilizing the library of foreign genetic elements interspersed between CRISPR sequences which act as the immune system memory of Bacteria and Archaea [35]. CRISPR loci, in general, consist of closely spaced direct repeats separated by short spacer regions of variable sequence. Spacer regions mostly correspond to sections of foreign plasmid or viral sequences which have been integrated. The CRISPR loci are found adjacent to the *casABC* operon. The fact that the *casABC* operon was significantly up-regulated by SlyA suggests that this regulator may contribute to viral resistance and immunity in *E. coli* K-12 MG1655.

Other transcripts that exhibited increased abundance in the presence of SlyA were associated with uptake and metabolism of phenylacetic acid (*paaA-K*), utilization of alkanesulfonates as alternative sulfur sources (*ssuEADCB*; divergently transcribed from the *elf* operon; see below), a cryptic galactosamine transport and catabolism system (*agaS-I*) and a 2-*O*-α-mannosyl-d-glycerate phosphotransferase and α-mannosidase (Table 2) [36–39]. Hence, it appears that SlyA plays a role in regulating systems that expand the repertoire of substrates utilized by *E. coli*. Increased abundance of the *mdtM* transcript suggests a role for SlyA in enhancing expression of this multi-drug transporter that confers resistance to ethidium bromide and chloramphenicol with mutants exhibiting attenuated growth at alkaline pH [40]. However, simple growth inhibition studies suggested that *slyA* expression led to increased sensitivity to chloramphenicol (growth yield after 6 h at 37 °C in LB broth was lowered to ~50 % by 2 µg ml^−1^ for the wild-type carrying the empty vector compared to 1 µg ml^−1^ for the wild-type carrying the *slyA* expression plasmid) rather than increased resistance, perhaps reflecting the complexity of the phenotype of the *slyA* expression strain.

Several of the SlyA-regulated operons code for proteins involved in membrane function. In *S. enterica* serovar Typhimurium, the majority of genes affected by SlyA encode proteins associated with the bacterial cell envelope and are important for virulence and survival within murine macrophages. Although it has been previously shown that the majority of genes regulated by SlyA in *S. enterica* serovar Typhimurium are not present in *E. coli* K-12 [12, 15], a similar propensity for cell envelope proteins being regulated by the *E. coli* SlyA was evident here. Of the 30 operons that exhibited altered expression in SlyA-expressing bacteria, 13 (43 %) were associated with cell surface/membrane functions (Table 2).

The *gspC-O* operon is a cryptic membrane-associated, H-NS-repressed, transcription unit that was up-regulated by SlyA (Table 2). The *gspC-O* operon encodes a type II secretion system for the export of endogenous proteins and formation of structural elements of the Gsp secreton, which is thought to facilitate the export of the endogenous endochitinase ChiA, a product of another H-NS silenced gene [41, 42].

Among the transcripts with increased abundance in the SlyA over-producing strain were four cryptic operons (*elfADCG-ycbUVF*, *sfmHF*, *yehDCBA* and *yadN*) encoding fimbrial-like adhesins (Table 2). These four operons were among seven putative chaperone-usher fimbrial systems shown to be poorly expressed under laboratory conditions by Korea *et al.* [43]. Nevertheless, when these operons were individually expressed by placing them under the control of a constitutive promoter, six were shown to be functional and expression of the *elf* (*ycb*), *yad* and *yeh* operons resulted in enhanced biofilm formation on abiotic surfaces, whereas *sfm* promoted binding to eukaryotic cells [43]. Moreover, all four operons were repressed by H-NS. The increased abundances of the *elf*, *sfm*, *yad* and *yeh* transcripts upon expression of SlyA are consistent with the cryptic status of these genes under normal laboratory conditions, and suggest that these chaperone-usher fimbriae are functional under environmental conditions that enhance *slyA* expression such that SlyA can operate as an H-NS antagonist (Table 2).

### SlyA over-production is associated with enhanced biofilm formation

**Fig. 2.**
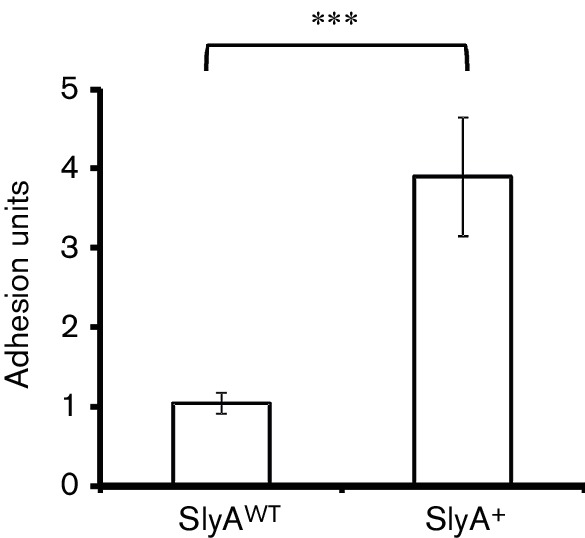
Biofilm formation by *E. coli* K-12 MG1655 is enhanced by elevated *slyA* expression. Wells containing M9 minimal medium with 20 % (w/v) glucose as a carbon source were seeded with 1 : 10 inocula of overnight cultures and incubated at 37 °C for 16 h. The OD_600_ of the planktonic bacteria was measured before a biofilm assay was carried out. Values shown are the mean and sd (*n*=12) and *** denotes *P*≤0.00001 in a Student's *t*-test.

The observation that SlyA increased transcription of four cryptic fimbrial-like adhesins suggested that the SlyA over-producing strain should exhibit enhanced biofilm production. This was tested using static cultures of *E. coli* K-12 MG1655 transformed with pET28a (control) or the *slyA* expression plasmid pGS2468 under conditions that mirrored the transcript profiling experiment. The data showed a fourfold increase in biofilm formation when *slyA* was over-expressed, consistent with the transcript profiling data (Fig. 2).

### Identification of new *E. coli* K-12 MG1655 operons that are directly regulated by SlyA

The changes in transcript profiles that were observed upon over-production of SlyA could have resulted from direct interaction of SlyA with the promoter regions of the corresponding genes or indirectly via SlyA-regulated factors. For example, one of the genes up-regulated upon SlyA over-production, *leuO*, encodes a transcriptional regulator that, like SlyA, operates by antagonizing H-NS regulation [44, 45]. Of the 27 transcripts that were increased in abundance when SlyA was expressed in *E. coli* K-12 MG1655, 14 (52 %) were associated with LeuO binding sites identified in the SELEX-chip study of Shimada *et al.* [44]. This strong correlation could arise from (1) the positive effect of SlyA on the expression of *leuO* resulting in an increase in expression of the entire LeuO regulon, i.e. indirect regulation by SlyA, or (2) SlyA and LeuO have overlapping regulons as a consequence of the fact that they both operate by antagonizing H-NS-mediated repression. To further investigate the extent of direct SlyA-mediated regulation in *E. coli* K-12 MG1655, binding of SlyA to 10 promoter regions was examined by EMSA.

**Fig. 3.**
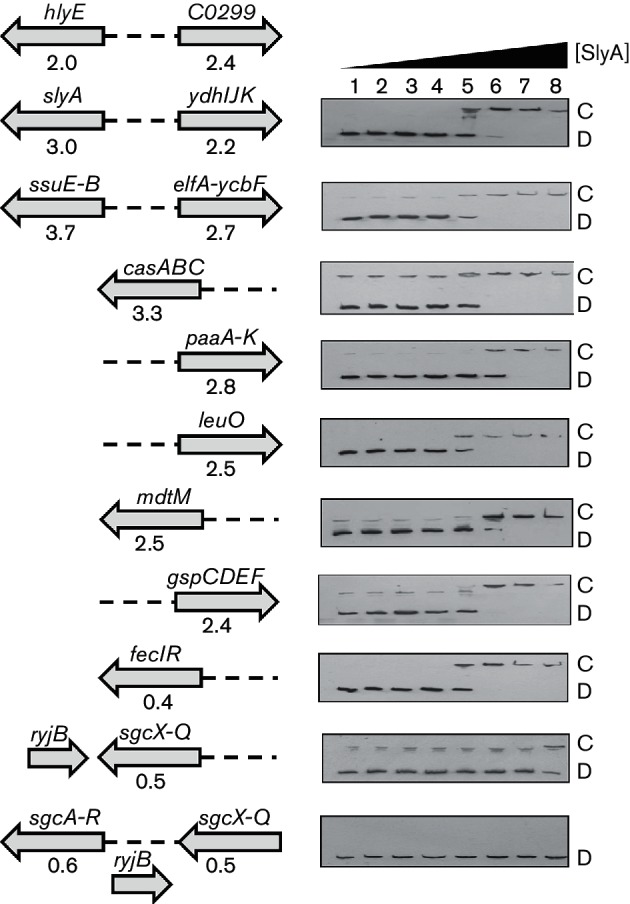
EMSA showing specific binding of SlyA to intergenic regions of selected operons. The dashed lines in the diagrams on the left indicate the DNA regions used in EMSA shown on the right. The arrows indicate the polarity of the genes (names above the arrows). The numbers below the arrows representing genes are the fold changes in transcript abundance observed upon over-production of SlyA (Table 2). SlyA binding to the *hlyE-C0299* intergenic region has been reported previously [20]. For EMSA, biotin-labelled intergenic DNA was prepared as described in Methods. Labelled DNA was incubated with increasing concentrations of purified SlyA protein, and protein/DNA complexes were separated by electrophoresis on native polyacrylamide gels. Lanes 1–8 : 0, 1, 5, 10, 50, 100, 200 and 500 nM SlyA. The locations of the free DNA (D) and the SlyA/DNA complexes (C) are indicated. Note that binding at the *sgcX* upstream region was only evident at the highest SlyA concentration tested, and the complex (C) was located close to a contaminating DNA species.

Among the transcripts differentially regulated by over-production of SlyA, there were three arranged as divergent operons (Fig. 3). Binding of SlyA at the *hlyE-C0299* intergenic region was shown previously (Fig. 3) [20]. Two other examples of SlyA-activated divergent operons (*slyA-ydhIJK* and *ssuE-B-elfADCG-ycbUVF*) were shown to bind SlyA in EMSA (Fig. 3). Furthermore, SlyA bound at the *casA*, *fecIR*, *gspCDEF*, *leuO*, *mdtM* and *paaA-K* promoters (Fig. 3). The *K*_d(app)_ values for SlyA binding at these promoters were similar, at ~50–100 nM. These experiments indicate that these operons are likely to be directly regulated by SlyA. The *sgcXBCQ-sgcAER* genes are separated by an sRNA *ryjB* on the opposite DNA strand (Fig. 3). It is suggested that the *sgcXBCQAER* is a single transcription unit, but there is no high-quality evidence to support this suggestion [46]. Therefore, both the region upstream of *sgcX* and the intergenic region between *sgcQ* and *sgcA* were used in EMSA with the SlyA protein. No specific interaction was observed with the region upstream of *sgcA* but interaction, albeit weaker than that observed for the promoter regions analysed above, was observed when the DNA upstream of *sgcX* was tested (Fig. 3). These observations suggest that *sgcXBCQAER* is a single SlyA-repressed transcription unit.

The EMSA experiments indicate that SlyA binds P*ssu*, P*cas*, P*paa*, P*elf*, P*leuO* and P*gsp*, all of which are promoter regions of genes or operons proposed to be part of the LeuO regulon (Table 2). This suggests that, perhaps because of the similarity in their mode of action, i.e. antagonizing H-NS repression, the SlyA and LeuO regulons substantially overlap such that, upon activation by their respective signals, a similar transcriptional response is elicited.

A consensus binding site (TTAGCAAGCTAA) for the *S. enterica* serovar Typhimurium LT2 SlyA protein was proposed based on footprinting and a limited SELEX analysis [10]. This consensus was further analysed by site-directed mutagenesis, which suggested the consensus sequence TTAN_6_TAA [9]. All the DNA fragments that bound *E. coli* SlyA in EMSA (Fig. 3) possessed DNA sequences similar to the previously proposed consensus sequences (Table 3). Site-directed replacement amino acid residues of *S. enterica* serovar Typhimurium LT2 SlyA identified 16 locations that impaired DNA binding [9]; all these amino acids are conserved in the *E. coli* SlyA protein, suggesting that these closely related proteins recognize similar DNA motifs.

**Table 3.** Candidate SlyA binding sites within the DNA fragments used for EMSA analyses Sequences shown are those with the greatest similarity to the previously proposed consensus for the *S. enterica* serovar Typhimurium LT2 SlyA protein (Haider *et al*. [9]; TTAN_6_TAA). Where more than one possible site was present, those with the greatest similarity to the consensus sequence TTAGCAAGCTAA proposed by Stapleton *et al*. [10] are shown. Locations of sites are given as the number of base pairs from the start codon of the specified gene to the centre of the proposed binding site.

Promoter region	Possible SlyA binding sites	Location of site relative to start codon
P*casA*	**TTA**TTGAAT**TAA**	100 bp upstream of *casA*
P*ssuE/elfA*	**T**C**A**GGATGA**TAA**	8 bp upstream of *elfA*
P*gspC*	**TTA**TATTAG**TAA**	79 bp upstream of *gspA*
P*paaA*	**TTA**AATCGCG**AA**	239 bp upstream of *paaA*
	**TTA**TAAAAA**TA**G	136 bp upstream of *paaA*
	**TTA**CTTAAC**TA**T	81 bp upstream of *paaA*
P*sgcX*	**TTA**TGCTGGG**AA**	336 bp upstream of *sgcX*
	**TT**TCAACCA**TAA**	188 bp upstream of *sgcX*
P*fecI*	**TTA**GAAAAAC**AA**	109 bp upstream of *fecI*
P*slyA*	**TTA**GCAAGC**TAA**	22 bp upstream of *slyA*
	**TTA**GATTAA**TAA**	161 bp upstream of *slyA*
P*leuO*	**TTA**ATGCAT**TAA**	305 bp upstream of *leuO*
	**TTA**AATATA**TAA**	297 bp upstream of *leuO*
P*mdtM*	**T**A**T**ACACCT**TAA**	249 bp upstream of *mdtM*

### Concluding remarks

SlyA proteins have been shown to play important roles in regulating gene expression in a wide range of bacterial species. The most common mechanism for SlyA-mediated activation of gene expression is through antagonism of H-NS repression. Here, transcript profiling has revealed the breadth of the SlyA regulon (directly and indirectly regulated genes) in *E. coli* K-12 MG1655 cultures grown under precisely controlled conditions such that any potential effects associated with changes in growth rate/growth phase could not confound the interpretation of the data obtained. Enhanced transcript abundance for several cryptic fimbrial operons in a SlyA over-producing strain and an over-representation of H-NS-repressed genes were consistent with the current model of SlyA-mediated gene activation. The SlyA protein was shown to bind at nine intergenic regions controlling the expression of 11 operons, thus expanding the number of known directly SlyA-regulated genes in *E. coli* MG1655 from 2 to 13.
